# Quantifying the impact of PM_2.5_ and associated heavy metals on respiratory health of children near metallurgical facilities

**DOI:** 10.1007/s11356-016-6734-x

**Published:** 2016-04-26

**Authors:** Daniel Dunea, Stefania Iordache, Hai-Ying Liu, Trond Bøhler, Alin Pohoata, Cristiana Radulescu

**Affiliations:** Valahia University of Targoviste, Aleea Sinaia no. 13, RO-130004 Targoviste, Romania; Norwegian Institute for Air Research - NILU, Instituttveien 18, PO Box 100, NO-2027 Kjeller, Norway

**Keywords:** Fine particulates, Environmental mapping system, Wheezing, Immunoglobulin E, Eosinophil count

## Abstract

The aim of this study was to link the concentrations of particulate matter with an aerodynamic diameter below 2.5 μm (PM_2.5_) and associated heavy metals with occurrence of wheezing and hospitalizations due to wheezing in 111 children who live near metallurgical plants in Targoviste City, Romania. A group of 72 children with high levels of immunoglobulin E (IgE) and eosinophils, as well as frequent wheezing episodes, was geolocated on digital thematic maps. Monitoring campaigns and medical assessments were performed over two consecutive years (2013–2014). The multiannual average concentrations of PM_2.5_ ranged from 4.6 to 22.5 μg m^−3^, up to a maximum value of 102 μg m^−3^. Significant correlations (*p* < 0.01) were observed between the locations of the children with respiratory issues and the PM_2.5_ multiannual average (*r* = 0.985) and PM_2.5_ maximum (*r* = 0.813). Fe, Ni, Cd, and Cr were the main marker elements of the emissions from steel production and metal-working facilities in the Targoviste area. The results support the hypothesis that increased PM_2.5_ levels directly influence wheezing symptom and asthma attacks in the analyzed group. IgE, eosinophils, and wheezing episodes may be considered key indicators with which to evaluate the adverse effects of PM_2.5_ air pollution on children’s health.

## Background

In the last decade, many residential areas throughout the world have been affected by fine particulate matter with an aerodynamic diameter below 2.5 μm (PM_2.5_), which disturbs air quality and favors the propagation of higher respiratory morbidity levels and numerous clinical symptoms, especially in infants and small children (Henschel et al. 2012; Pope and Dockery 2006; Ward and Ayres 2004). These age groups, infants and children, are at risk to suffer from adverse health effects due to air pollution (Bilenko et al. 2015; Pohoata and Buzatoiu 2014; Rivas et al. 2014; WHO 2013). Air pollution episodes have been linked to asthma attacks in children with asthma, which may induce serious complications, leading to respiratory failure, if not properly treated (Anderson et al. 2013; Buonanno et al. 2013; Hay et al. 2014; Landrigan and Etzel 2014). Such episodes are characterized by abnormally high concentrations of air pollutants during prolonged periods, often due to low winds, absence of rain, and temperature inversion (Dunea et al. 2015). Children who live near intense traffic are more susceptible to developing asthma (Gasana et al. 2012; Gowers et al. 2012; Newman et al. 2014).

A progressive increase in the number of respiratory diseases has been reported in Romanian urban areas, including infants with recurrent wheezing, asthma, and rhinitis in preschool; this association has been confirmed by the recent studies (Craiu and Stan 2014). Statistical reports show a 10–15 % annual increase in the diagnosis of asthma in Targoviste City, which is located near metallurgical facilities. In a high number of cases, diagnosed with asthma was found to be accompanied by allergic rhinitis and atopic dermatitis (chronic urticaria). The incidence of allergic diseases is significantly increasing, with one out of three children having a family history of allergy (Pohoata and Buzatoiu 2014).

Particulate matter (PM) mixtures have different physical and chemical characteristics, which are associated with an array of adverse effects on human health (Amodio et al. 2013; Mölter et al. 2015; Querol et al. 2007; Radulescu et al. 2015). The combined use of the inventory of pollutant emissions, the number of children with respiratory diseases, and the scenarios that result from the utilization of dispersion models that complement and/or supplement in situ measurements facilitates the identification of critical areas and their prioritization (Iordache and Dunea 2013; Mohan et al. 2011; Puliafito et al. 2003). Spatiotemporal and qualitative characteristics of PM_2.5_ data are essential for supporting the epidemiological studies to consolidate the knowledge regarding the effect of particle size and chemical composition on children’s health (Baltrenaite et al. 2014; EEA 2014; Neuberger et al. 2004; Olsen et al. 2014). Children present an increased risk from the effects of air pollution due to their lung immaturity and sensitive immune system (Bateson and Schwart 2008; Sheffield et al. 2015). Our hypothesis is that the levels of allergic indicators, i.e., immunoglobulin E (IgE) and eosinophils in children, may facilitate the assessment of the PM_2.5_ effects on respiratory health. IgE plays an important role in mediating allergic reactions that occur after exposure to allergens in susceptible (atopic) individuals (Pohoata and Buzatoiu 2014). Eosinophils (granulocytes) are actively involved in various inflammatory processes and are typically present in high numbers in allergic diseases and other medical conditions (Liu et al. 2011).

This study aimed to link airborne fine particulate matter levels and their heavy metal content with wheezing occurrence in children at a spatiotemporal scale in urban areas of Targoviste, Romania, by focusing on the exposure of the assessed population located near metallurgical plants. This case study provides a framework for detailed environmental exposure and epidemiological assessments regarding asthma causality due to PM_2.5_ air pollution.

## Materials and methods

**Fig. 1:**
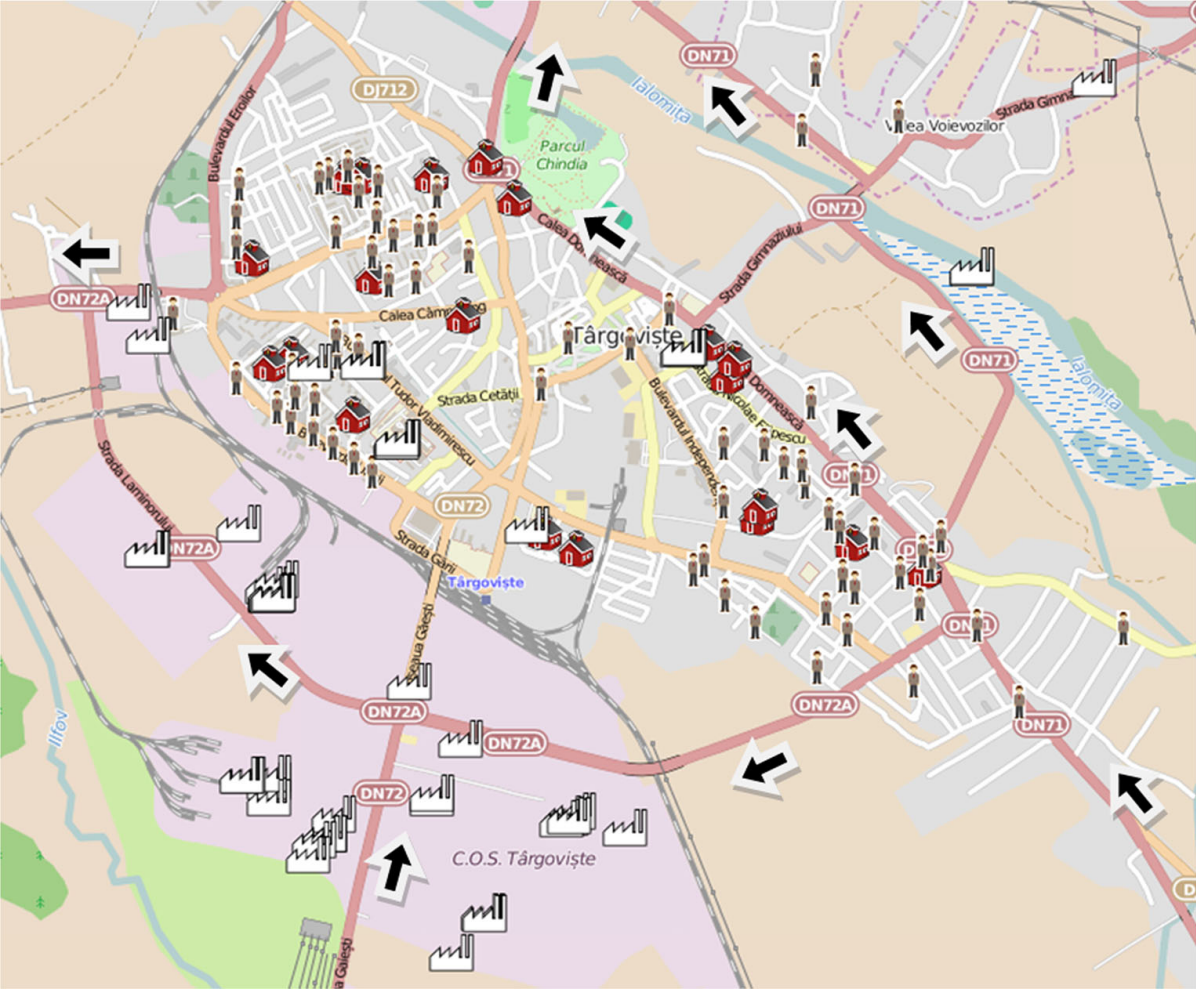
Spatial distribution of main stationary sources (metallurgical industry in the south), schools and kindergartens, and the incidence of respiratory diseases with wheezing episodes in Targoviste during the study period of 2 years; *arrows* denote streets with heavy traffic

Quantifying the health impact of PM pollution within an area is facilitated by the elaboration of a logic diagram of monitoring activities and an appropriate experimental design protocol (Fig. 1). The classification of source apportionments and the evaluation of a control strategy also require the chemical speciation quantification of the PM fraction of interest (Chow and Watson 1998).

The conducted analysis had the following sequence of steps: (1) establishing the health database and identifying subjects according to their diagnoses, (2) geocoding the subjects based on home address and medical records, (3) spatial grouping of subjects, (4) analyzing the location of major emission sources: point sources, roads, diffuse sources, etc., (5) modeling the PM_2.5_ concentrations in the city, (6) establishing a monitoring plan based on dispersion modeling results selecting relevant monitoring locations, (7) assessment of heavy metal content in PM_2.5_ samples, (8) looking at the relationship between location a subject is likely to be and pollutant concentrations at location, and (9) associating respiratory diseases with PM_2.5_ presence and heavy metals content.

Monitoring campaigns of PM_2.5_ and associated heavy metals were performed over two consecutive years (2013–2014) at 10 representative monitoring points in the Targoviste urban area of Romania (latitude 44° 56′ N, longitude 25° 26′ E, altitude 280 m). The city has 73,964 permanent residents (2014 census). The schools, kindergartens, and nurseries of Targoviste City had registered 6959 children for preschool and primary education (approximately 9.5 % of the total population), with ages between 0 and 10 years. Table 1 summarizes the characteristics of the population analyzed in the present study.

**Table 1:** Description of the study population in Targoviste City

Indicator	Descriptor
Study period	Between 01.2013 and 12.2014
Date of birth	Between 01.2004 and 12.2012
Age groups	Total of 111 children: infants – 0–2 years (21), preschoolers – 3–5 years (55), school children – 6–10 years (35)
Gender	60 males (54 %), 51 females (46 %)
Grouping by home address in a region of the city (total of children)	Group A (61), Group B (20), Group C (30)
Grouping by home address in a region of the city (sensitive children)	Group A (38), Group B (7), Group C (27)
Selection criteria for sensitive children	Number of wheezing episodes; eosinophil count; immunoglobulin E (IgE) serum level; response to inhalation medication
Medical records	Numbers of wheezing episodes and hospitalizations were counted starting with a child’s first consultation/admission in hospital during the study period; blood test indicators of each child were averaged on the study period

### Point sources and particulate matter dispersion in the area

A metallurgical plant for specialty steel production and several metalworking facilities (e.g., stainless steel strips, sheets, small profile components and wire rods; non-grain-oriented electrical steel; chrome bars and skived and roller-burnished tubes) are situated south, near the city limits. A company that produces rigs is located in the city center; in the past, a steel foundry, a forge, and other metallurgical facilities were present. Some of these facilities were dismantled, and the buildings were demolished. A coal thermal plant, which stopped functioning in 2009, and some chemical point sources are located 7 km northwest of the city.

The AERMOD model, which is approved by the US EPA for the majority of regulatory air dispersion systems, was used with the *BREEZE*® AERMOD 7.9 software (Trinity Consultants, Dallas, TX, USA), which enables the simulation of plume rise and plume penetration for elevated inversions and improved computation of vertical profiles of wind, atmospheric turbulence, and air temperature. Meteorological input data consisted of extensive time series (1961–2013) extracted from ROCADA, a Romanian daily gridded climatic dataset (Birsan and Dumitrescu 2014). The model provided the PM_2.5_ concentration at receptor levels for various scenarios based on emission regime, PM_2.5_ emission rates (g s^−1^), and time intervals. The modeling results facilitated the optimal positioning of PM_2.5_ sampling points.

### Monitoring plan and sampling procedure

The sampling points were established using a top-down approach (i.e., previous measurements, data analysis, and receptor modeling based on emission source profiles) that produced a quasi-radial spatial disposal in relation to the shape of the city allowing a suitable assessment of PM_2.5_ at city level. Other criteria included the proximity to schools, kindergartens, and playgrounds. Only one station (RO030401, industrial type) of the national authority for air quality monitoring is located in the city, having a PM_10_ optical analyzer that has been faulty many times determining a lack of observable data. Consequently, we used two optical portable monitoring systems, i.e., Dusttrak^TM^ DRX 8533EP with an environmental enclosure (www.tsi.com) and Casella® Microdust Pro (www.casellasolutions.com), which provided the PM_2.5_ concentrations at the designated sampling points. The flow rate of the external pump was 3 L min^−1^ for both instruments. Particles were collected on 37-mm quartz fiberglass filters (QM-A Whatman, Maidstone, Kent, UK) in specific cassettes. Blank filters were weighed on an analytical microbalance and were labeled prior to use. Instruments were placed in the designated outdoor locations on tripods at heights between 1.30 and 1.50 m and away from obstructions that may affect wind currents. The sampling time was 1 h at each point to ensure a sufficient PM_2.5_ mass for heavy metal detection, and the log interval was 10 s. Optical instruments were moved to the next point in a random sequence for each campaign to determine the PM_2.5_ levels on a city scale for different hours of the day. Measurements were done particularly during the “rush” hours (7.00–9.00 a.m. and 12.00–2.00 p.m.) and according to the potential regular outdoor program of children (3.00–6.00 p.m.) allowing the actual exposure assessment of the studied population. Two monitoring campaigns were performed each month depending on the rainfall regime (measurements were performed after a minimum of 3 days after a rainy day because precipitations and elevated relative humidity of air have been found to reduce the PM concentrations). We obtained 48 PM_2.5_ time series for each point and, implicitly, 48 discs for the analysis of heavy metals. These data were averaged and correlated with the medical parameters. A heated inlet with an auto zero module mounted on Dusttrak^TM^ was utilized on cold and misty days. A circular area of representativity (±15 %) with a radius of 500 m was considered for each sampling point. The incidence of respiratory diseases was counted in each of these areas, and Pearson’s correlation coefficients were computed among variables.

### Elemental analysis of PM_2.5_ content

The collected filters were analyzed using a graphite furnace atomic absorption spectrophotometry (GFAAS) technique to determine the heavy metal composition of captured fine particulates. Samples were collected in accordance with a manual for sampling and chemical analysis (EMEP 2001). Each filter was stored in Petri dishes in desiccators to complete the conditioning process. The samples were digested on a hot plate using TOPwave microwave-assisted pressure digestion. Acidified extracts were filtered after digestion through a Whatman 41 filter paper, which was previously rinsed with 1 % HNO_3_. The metal concentrations were determined using a ZEEnit 700 P spectrometer, which combines a furnace with reliable deuterium and Zeeman background correction for optimal results. All chemical reagents were of analytical grade. Deionized water (resistivity of 18.2 MΩ cm^−1^) was obtained with a Milli-Q System (Millipore, Bedford, MA, USA). Nitric acid (high purity, Merck) was used for the preparation of the blank (1 % nitric acid). All sample containers, auto-sampler cups, and other materials were washed with water, soaked in 10 % v v^−1^ nitric acid for 24 h, and rinsed with deionized water prior to use. Quantification was performed by standard curves. The metal calibration curves showed adequate linearity over the concentration range (0.1 to 10.0 mg L^−1^) with *R*^2^ correlation coefficients in the range of 0.996 to 0.999 (Radulescu et al. 2015). The analytical curves for each analyzed element were prepared using a stock standard solution (Merck). In this experiment, the LODs (lowest concentration that can be detected with GFAAS) of the analyzed elements were established using the calibration data. The measurements were performed in triplicate. The LOD values were calculated from the equations: (3 × SD/*α*), where SD is the standard deviation of the blank and *α* is the angular coefficient of the analytical curve. Some LOD sample values were expected to be less than the estimated LOD. For example, for lead (Pb), the estimated LOD was 0.2 μg L^−1^ per sample, which was similar to the expected LOD value. The LOD for the cadmium (Cd) analysis by GFAAS was 0.5 μg L^−1^. Standard reference materials (i.e., NIST SRM 1648a, Urban Particulate Matter) were used to verify the accuracy and traceability of the method. The relative standard deviation (RSD) of the standard was 0.36 %, the RSD of the samples ranged from 1.2 to 2.4 %, and the recovery rate ranged from 94.2 to 101.5 %. The concurrent interference was low.

### Medical database

The diagnosis of children with asthma, particularly in the preschool period, is primarily based on a clinical judgment and evaluation of specific symptoms and quality of life. Although wheezing is the most common sign of asthma, some children also present with recurrent cough and prolonged expiration (Giovannini et al. 2010).

The medical database was developed with the support of the Targoviste Emergency Hospital, the Local Public Health Direction, and several pediatric doctors. The database contains as main fields the child’s anonymous identification code, age, the number of wheezing episodes, the number of asthma attacks (with hospitalization), the response to inhalation medication, the medication controller specifications, the eosinophil count, the immunoglobulin E (IgE) serum level, the residential address, and the school/kindergarten address (Dunea et al. 2014).

The number of episodes was counted starting with a child’s first consultation or emergency department visit during the assessed time interval (January 2013–December 2014). Blood test indicators of each child were averaged on the study period. The main factor for selecting the respiratory illnesses that were linked to atmospheric pollution for children was the *wheezing* symptom. Consequently, the children’s illnesses of interest in our study included the occurrence of several respiratory diseases potentially determined or aggravated by the atmospheric pollution, classified according to the International Classification of Diseases ICD-10 - WHO (2016), as shown in Table 2. The age variable was described using three categories: infants (I)—0–2 years (21 children), preschoolers (PS)—3–5 years (55 children), and school children (S)—6–10 years (35 children).

**Table 2:** Diagnoses potentially determined or aggravated by the atmospheric pollution recorded in the analyzed group of children and classified according to the International Classification of Diseases ICD-10, WHO (2016)

Diagnosis code	Diagnosis name
J21.9	Acute bronchiolitis, unspecified
J44.8	Chronic bronchitis: asthmatic (obstructive) NOS
J45.0	Predominantly allergic asthma
J45.9	Asthma, unspecified
J46	Status asthmaticus
J84.9	Interstitial pulmonary disease, unspecified
J96.0	Acute respiratory failure
J20.9	Acute bronchitis, unspecified
J40	Tracheobronchitis NOS
J30.4	Allergic rhinitis, unspecified
J31.0	Chronic rhinitis NOS
R06.2	Wheezing (Abnormalities of breathing)

### Environmental mapping system

GPS measurements established the exact positions (WGS-84 reference system) of each sampling point, child’s address, schools and kindergartens, as well as the main point sources, which facilitated the development of the corresponding layers (Fig. 1) for digital map production in QGIS (www.qgis.org).

The collected datasets were used to obtain PM_2.5_ and heavy metals isolines of concentrations, which were overlapped on the specific layers of vulnerable receptors in urban areas (e.g., kindergartens, schools, and playgrounds) using GIS capabilities. Kriging interpolation was applied to obtain the specific isolines of PM_2.5_ concentration. Geospatial analysis techniques were used to establish the overlapping results between the distribution of particulate matter and the locations of affected children.

### Statistical analysis

Descriptive, associative, and comparative statistics of the recorded time series were analyzed using SPSS software (SPSS Inc., Chicago, IL, 2011). Pearson product moment correlation was applied to identify the strength of the linear relationship between the variables. The computation of multiple range tests (LSD) provided the statistical significance of comparisons between years and locations. Factor analysis (FA) was performed using principal component analysis (PCA) based on Varimax with Kaiser normalization (Amodio et al. 2013; Hooyberghs et al. 2005; Liu et al. 2012) to reduce the number of factors that explains the variability in air pollution and children’s medical records from Targoviste City. Rotation of the factor axes (dimensions) resulted in the initial extraction of factors provided simple and interpretable results. Consequently, the rotation allowed a more detailed analysis of the first FA results making the structure of loadings more explicit (Dunea and Iordache 2015; Oakes et al. 2014). The input matrix began with 111 objects (number of children) by 7 variables (wheezing episodes, number of hospitalizations, levels of IgE, eosinophils, age, gender, and pollution area). Three factors were selected based on the eigenvalues that satisfied the Kaiser criterion (>1). Eigenvalues describe the variance in the population accounted for by each factor. A factor with a low eigenvalue has a diminished contribution to the explanation of variances in the variables and may be ignored.

## Results and discussion

As a starting point, this study utilized the findings for a group of children between the ages of 0 and 10 years who may be directly affected by air pollution with airborne particulate matter that primarily originated from metallurgical processes, urban traffic, and domestic heating. The analysis considered the medical aspects of 111 children diagnosed with respiratory diseases associated with wheezing. Children’s respiratory symptoms that were potentially determined or aggravated by atmospheric pollution included upper respiratory airways symptoms (cough, rhinorrhea, and sore throat) and lower airways symptoms (dyspnea, wheezing, and thoracic pain).

The location where the symptoms were likely to occur was spatially described using the children’s addresses to assess the potential correlations with PM_2.5_ levels. The locations of the affected areas (kindergartens, playgrounds, and schools) where children might be exposed to PM_2.5_ levels were also added to the digital map to facilitate the establishment of critical areas. Figure 1 shows the spatial positioning of 72 children selected from a total of 111 children, who presented high levels of IgE (normal value <60 units/ml) and a high eosinophil count (normal value = 0.1–3 % from leukocytes), as well as wheezing episodes.

**Fig. 2:**
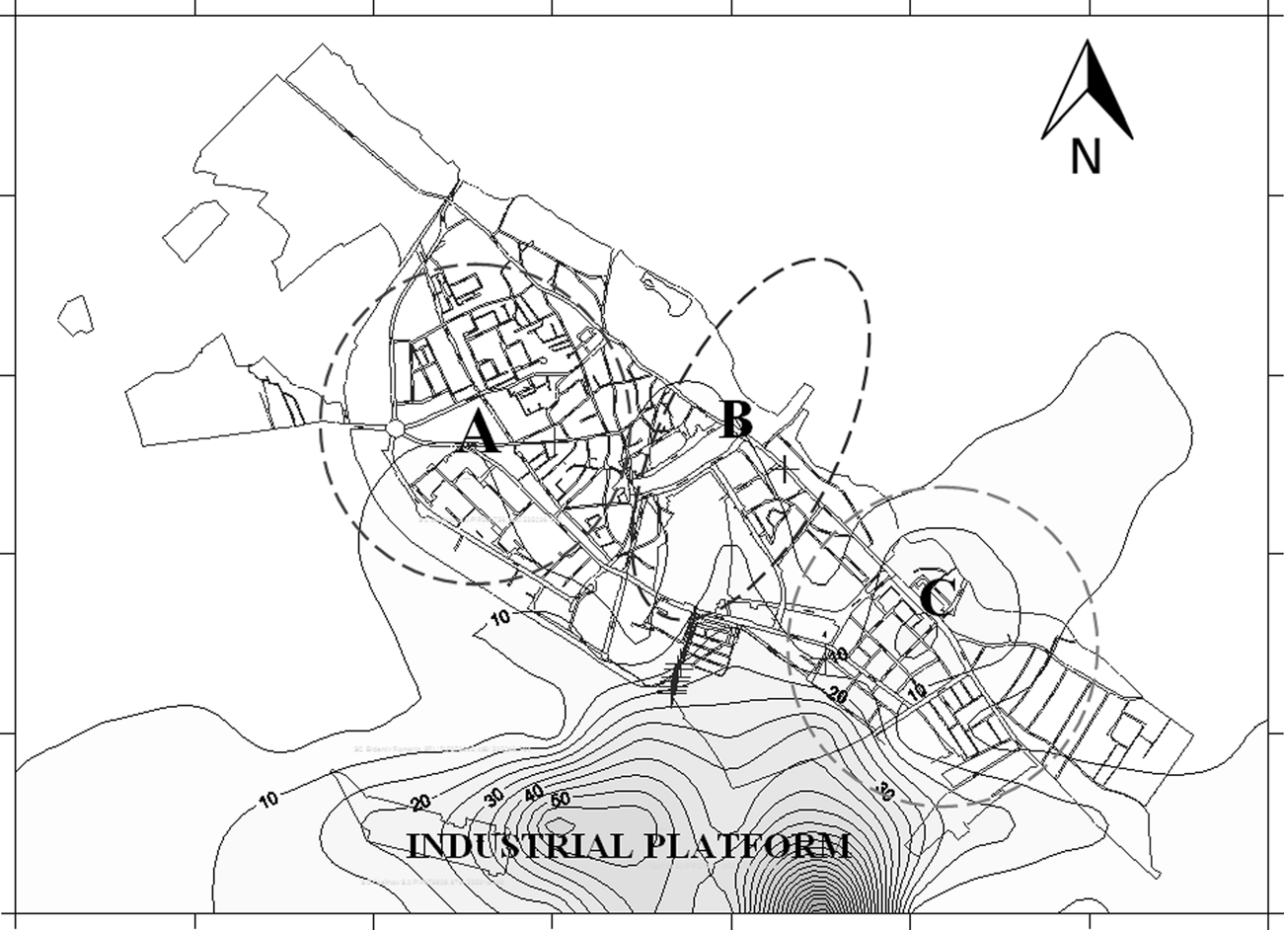
Dispersion of annual average concentrations of PM_2.5_ (μg m^−3^) from the stationary sources located in the industrial sector (predominantly metallurgical industry) in the Targoviste urban area; *dotted circles* represent areas with a high incidence of wheezing symptoms in children during the study period of 2 years

The spatial analysis revealed three groups of children: (A) northwest group—61 children, (B) center-northeast group—20 children, and (C) southeast group—30 children. The specific layer was included in the environmental mapping system after the selection of children who manifested an allergenic response: 38 children (A), 7 (B), and 27 (C), which were usually more sensitive to PM_2.5_ pollution (Figs. 1 and 2). The main pollution point sources and roads with heavy traffic were overlapped to provide a comprehensive image of the emissions’ impact on the sensitive receptors.

**Fig. 3:**
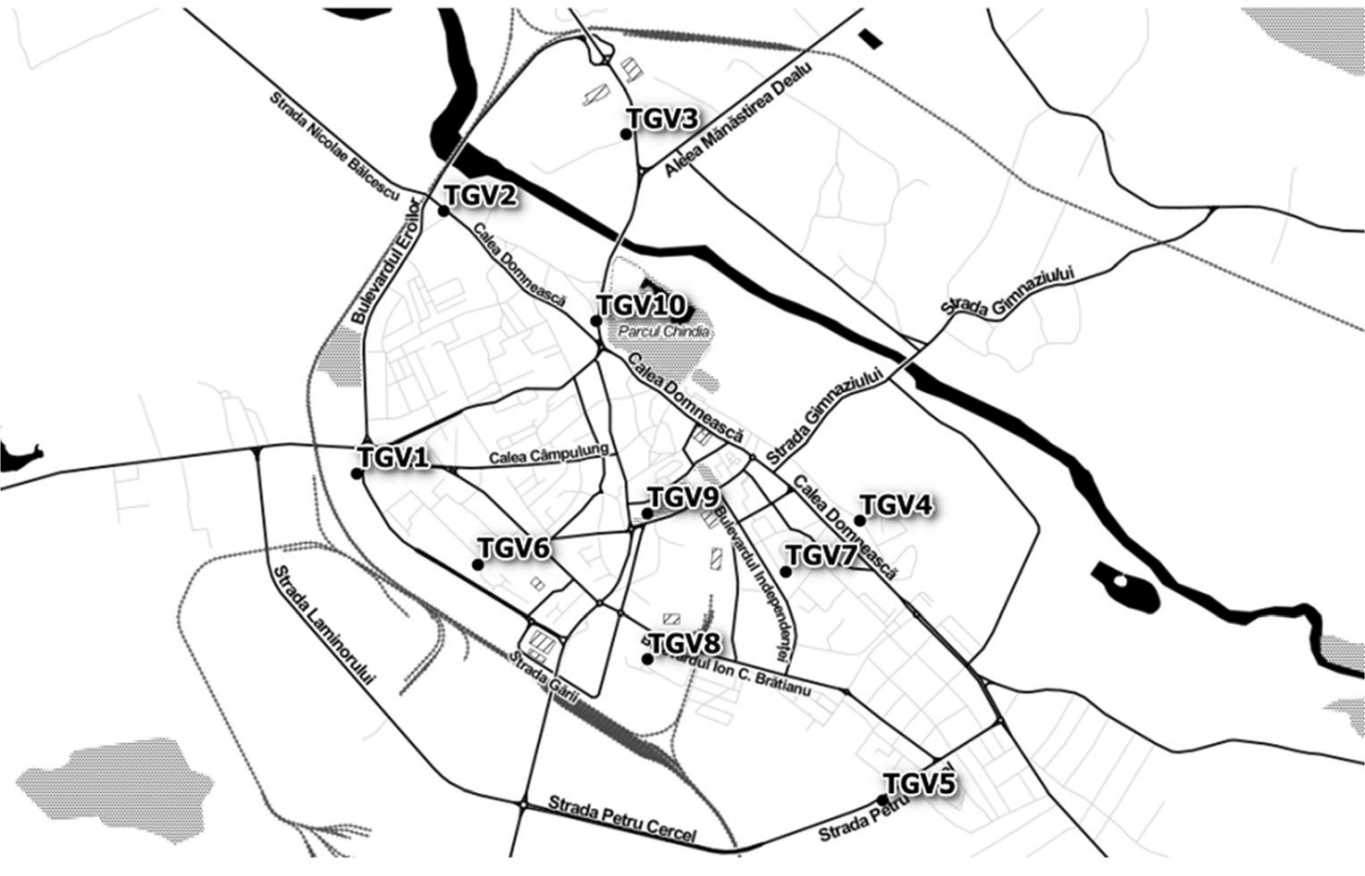
Distribution of the 10 PM_2.5_ sampling points in the Targoviste urban area; selection was based on a top-down approach and a quasi-radial spatial arrangement (TGV, identification code for Targoviste sampling points)

Model simulations were performed by grouping the stationary sources by cardinal directions, i.e., north, east, south, and west. The most important contribution of PM_2.5_ was estimated to originate from the industrial metallurgical facility located south of city. Figure 2 shows the average concentration highlighting that the location where group C is located was the most heavily impacted by PM_2.5_ air pollution (10–20 μg m^−3^ PM_2.5_). A monitoring plan was developed based on the receptor modeling results. Figure 3 presents the location of the PM_2.5_ sampling points that were established using a top-down approach.

Our results of an association between PM_2.5_ levels, meteorological factors, and an increased number of hospital admissions corroborate previous findings (e.g., Basagana et al. 2015; Neuberger et al. 2004).

### Geolocated study on selected groups of children

A geolocated study was developed by positioning each of the 72 children with high levels of IgE and eosinophils based on their residential address to link the airborne PM_2.5_ levels with the potential adverse health effects in children (Fig. 1). The majority of children had also experienced wheezing episodes. The group of 111 selected children who were vulnerable to air pollution consisted of 60 males (54 %) and 51 females (46 %) who were born between 2004 and 2012. The ranking of the age categories were as follows: 5 years (18 cases), 7 years (17), 2 years (14), 6 years (13), 4 years (13), 3 years (11), 8 years (8), 9 years (7), 1 year (7), and 10 years (3). The categories between 2 and 7 years accounted for 77.5 % of the group, which suggests that children in this age range are the most vulnerable to the occurrence of wheezing-related diseases (i.e., asthma attacks, bronchitis, and recurrent wheezing). More than 80 % of children with asthma exhibited distinct symptoms before the age of 5 years (Hay et al. 2014). The main symptoms experienced by the selected group were chest pain, prolonged cough, intolerance to physical effort, breathing difficulties with varying frequency and intensity, recurrent bronchitis, and pneumonia. Table 3 shows the descriptive statistics associated with the group of 111 children. Of particular interest were the numbers of children who exceeded the normal thresholds of IgE (56 children) and eosinophils (41 children). Many children presented with IgE values at the upper limit of the normal interval (e.g., mode was 60 units/ml). The statistical results during the study period of 2 years indicated median values of 7 wheezing episodes, 2 hospitalizations/child, 153 U/ml IgE, and 3.8 % for eosinophils, whereas the maximum values were 50 episodes, 10 hospitalizations/child, 2500 U/ml IgE, and 26 % eosinophils.

**Table 3:** Descriptive statistics of the indicators that are potentially related to the effect of PM_2.5_ on children’s health in Targoviste City (sample population = 111 children with recurrent wheezing between the ages of 0 and 10 years)

Indicator	Number of wheezing episodes	Number of hospitalizations	Immunoglobulin E (IgE) (U/ml)	Eosinophil count (%)
Valid N (cases)	72	53	72	68
Average	11.3	2.64	319.53	4.93
Median	7	2	153	3.85
Minimum	1	1	4.67	0.1
Maximum	50	10	2500	26
Std. deviation	10.87	1.63	488.46	4.97
Coeff. of var.%	96.21	61.71	152.87	100.87
Mode	3	2	60	0.8
Skewness	1.84	2.08	2.83	1.96
Kurtosis	3.03	6.86	8.36	5
Exceeding of normal threshold	–	–	56	41
Frequency of exceeding (%)	–	–	77.78	60.29

After performing the geolocation of children, a comparison of the geometrical means showed that the highest values of IgE (187.5 U/ml) and eosinophils (4.2 %) were recorded for the group A, followed by the group C (126.5 U/ml IgE and 1.6 % eosinophils) and the group B (66.6 U/ml IgE and 2.9 % eosinophils). No significant differences (*p* < 0.05) were observed when performing multiple range tests (LSD) between groups for wheezing episodes, IgE level, or eosinophil count. Higher values were recorded for all considered variables in the group A, which was located in northwestern Targoviste City. Another objective of the study was to determine whether the concentrations of PM_2.5_ are correlated with the spatial distributions of respiratory diseases in children.

### PM_2.5_ levels and spatial correlations with wheezing occurrence

**Fig. 4:**
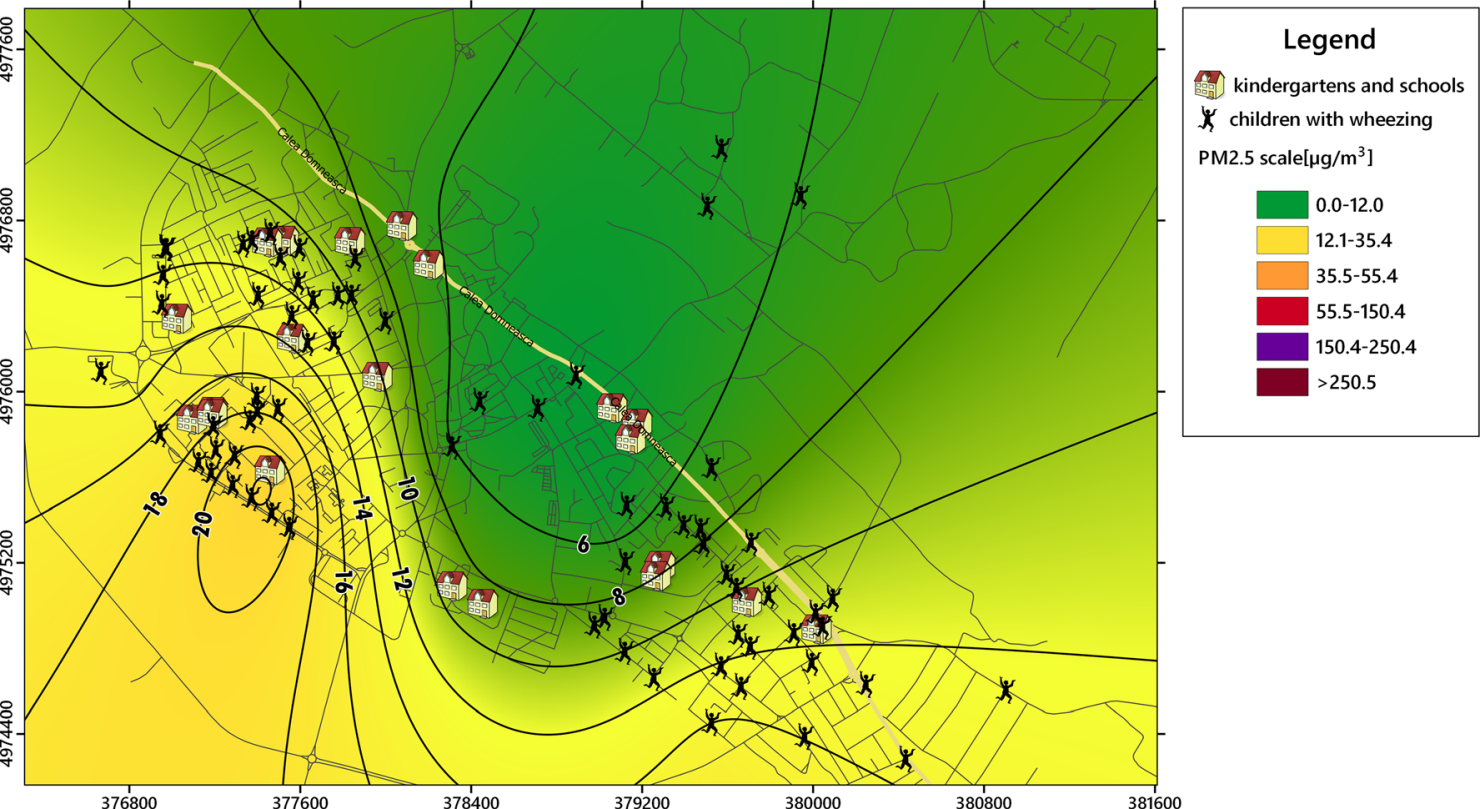
Isolines of PM_2.5_ annual average concentrations (μg m^−3^) recorded during the most probable hours of children’s outdoor program in Targoviste at 10 monitoring points using Kriging interpolation

**Fig. 5:**
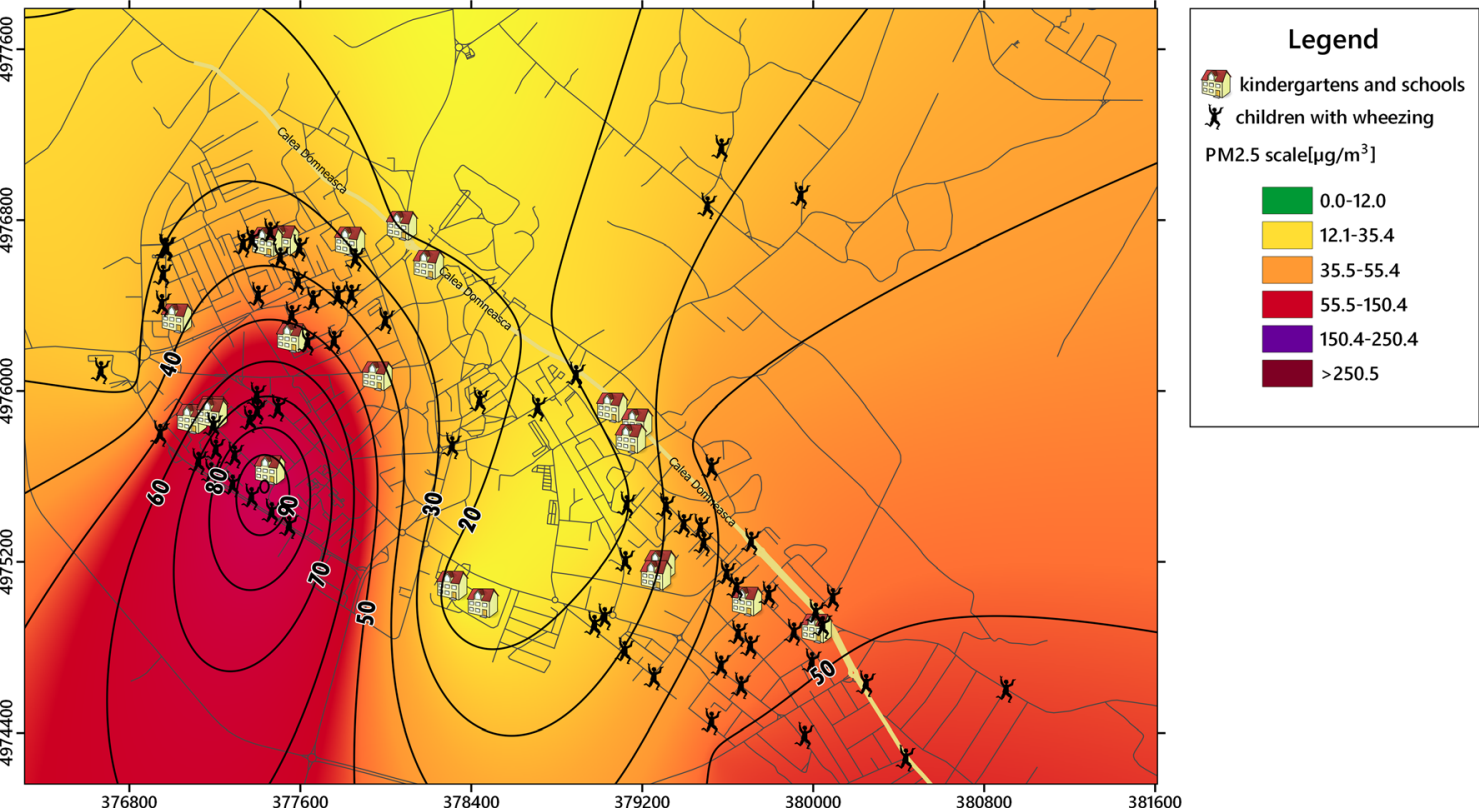
Isolines of PM_2.5_ annual maximum concentrations (μg m^−3^) recorded the most probable hours of children’s outdoor program in Targoviste at 10 monitoring points using Kriging interpolation

The PM_2.5_ multiannual average of measured concentrations ranged from 4.6 to 22.5 μg m^−3^ with a coefficient of variation (CV) of 57.3 %, and the maximum concentrations ranged from 13.1 to 102 μg m^−3^ (CV = 81.3 %), depending on the sampling point (Table 4). The average of the maximum absolute values was 187.1 μg m^−3^ (CV = 175.29 %). The thematic maps with isolines of concentrations showed high levels of PM_2.5_ in the western and northwestern parts of the city, which were correlated with intense heavy traffic and neighboring active industries from the northwest (Figs. 4 and 5). The impact of emissions generated by the southern metallurgical facility was less evident due to lower emissions because of the economic recession since 2009, which have affected the industrial production. In situ measurements showed a different pattern compared with the dispersion modeling results (Table 5), which revealed higher annual average concentrations (10–22.5 μg m^−3^) in the group A area; the concentrations of the group C area ranged from 6 to 14 μg m^−3^ (Figs. 4 and 5). The PM_2.5_ map showed that the group B area recorded the lowest concentrations (≤6 μg m^−3^). Highly significant correlations (*p* < 0.01) were observed between the locations of the children with high number of wheezing episodes and hospitalizations and the PM_2.5_ multiannual average (*r* = 0.985), PM_2.5_ maximum values (*r* = 0.813), and PM_2.5_ momentary peak values (*r* = 0.802). This spatial correlation supports the hypothesis that the respiratory health impact of increased PM_2.5_ concentrations was more pronounced in areas with presumptively higher exposure (e.g., playgrounds, schoolyards, etc.), having a direct influence on wheezing-related symptoms and asthma attacks in the analyzed group of children.

**Table 4:** Centralized results of the PM_2.5_ measurements (μg m^−3^) and corresponding heavy metal concentration (ng m^−3^) recorded in Targoviste City between 2013 and 2014 in 10 sampling points (Fig. 3)

Sampling point	Latitude WGS 84	Longitude WGS 84	PM_2.5_ average	PM_2.5_ maximum	PM_2.5_ Peak	Pb	Cd	Cr	Ni	Fe
1	44.92722	25.43891	13.16	27.63	70	2.11	0.25	0.06	0.87	5.83
2	44.93965	25.44471	8.45	24.63	110	2.27	0.24	0.04	0.82	4.17
3	44.9433	25.45695	7.15	13.14	28	2.57	0.24	0.07	1.16	3.91
4	44.92496	25.47263	6.03	41.60	130	0.99	0.11	0.02	0.75	3.10
5	44.91173	25.4742	15.41	51.10	212	0.83	0.09	0.01	0.54	3.19
6	44.92291	25.44697	22.55	102.00	1107	2.13	0.21	0.07	1.13	3.98
7	44.92257	25.46764	4.61	15.40	33	2.87	0.01	0.01	0.63	3.80
8	44.91839	25.45843	9.14	14.00	63	1.35	0.10	0.05	0.82	4.09
9	44.92533	25.45836	5.32	26.00	82	1.03	0.17	0.09	0.84	5.05
10	44.93444	25.45494	6.56	17.67	36	1.97	0.18	0.01	0.73	3.36
Average	–	–	9.84	33.32	187.10	1.81	0.16	0.04	0.83	4.05
Median	–	–	7.80	25.32	76.00	2.04	0.18	0.05	0.82	3.95
Coeff. of var. (%)	–	–	57.33	81.34	175.29	39.38	49.91	68.51	23.49	20.81

**Table 5:** Grouping of children with respiratory issues and PM_2.5_ monitored and modelled values (μg m^−3^) and corresponding heavy metal concentrations (ng m^−3^) in the urban areas of Targoviste City

Group^a^	Total number of children	Sensitive children	PM_2.5_ average	PM_2.5_ maximum	PM_2.5_ Peak	PM_2.5_ average (modelled) ^a^	Pb	Cd	Cr	Ni	Fe
A	61	38	11.7	36.1	268.6	7.2	2.3	0.2	0.1	1.0	4.4
B	20	7	7	19.2	60.3	8	1.5	0.2	0.1	0.8	4.2
C	30	27	8.7	36	125	12.5	1.6	0.1	0	0.6	3.4

Dispersion modeling was performed with the *BREEZE*® AERMOD 7.9 software

^a^Group A (northwest of city); group B (center-northeast group); group C (southeast of city)

### Heavy metal concentration of PM_2.5_

The laboratory analyses indicate that PM_2.5_ contained the following concentrations of heavy metals (multiannual averages) in descending order: Fe (3.1–5.8 ng m^−3^, CV = 20.9 %), Pb (0.8–2.8 ng m^−3^, CV = 44.7 %), Ni (0.5–1.16 ng m^−3^, CV = 23.5 %), Cd (0.01–0.25 ng m^−3^, CV = 49.9 %), and Cr (0.01–0.09 ng m^−3^, CV = 68.59 %). PM_2.5_ composition was similar to the rankings observed in other studies performed near steel-related sites (Dai et al. 2015; Querol et al. 2007; Taiwo et al. 2014) as shown in Table 4. The results are consistent with the concentrations of heavy metals recorded in certain urban US areas for corresponding mass concentrations (Chow and Watson 1998).

Our findings show that nickel (Ni) concentrations were more consistent compared with other metals. No statistical significances between children’s locations and any determined metal concentrations (*r* = −0.007–0.21) were observed. This result suggests that other air pollutants of concern in the ambient air and/or compounds (organic compounds and salts) in PM_2.5_ may have an immediate adverse effect on children’s respiratory health.

Lead (Pb) is present in paved road dust due to deposition from previous emissions of leaded-gasoline vehicle exhaust (Lu et al. 2014; Wei et al. 2015). The highest concentrations were recorded in areas with intense traffic (i.e., central market and city exits of the main roads; see points TGV 7, 3, 2 and 1). Cadmium (Cd) primarily originates from steel production, pigment facilities, and tire wear (Tian et al. 2012). The highest Cd concentrations were observed in the western and northwestern sections of the city (i.e., points TGV 1, 2, 3, and 6). Chromium (Cr) occurs in soluble forms from fossil fuel combustion and vehicle emissions (Abuduwailil et al. 2015). The highest values were recorded in the city center and toward the west and northwest (points TGV 9, 6, 3, and 1). Nickel (Ni) was emitted in the area from steel production and coal/oil combustion. The highest values were recorded at points TGV 3, 1, 9, 8, and 2 due to dust resuspension and residential heating. Iron (Fe) was the most abundant element in PM_2.5_; it primarily originated from steel dust accumulation in the area. High concentrations of this element also occurred in suburban areas. The highest multiannual averages of Fe concentrations were observed at the TGV 1, 9, 2, and 8 sampling points. The bivariate relationships between metal concentrations were only significant for the following pairs: Cd–Ni (*r* = 0.69; *p* < 0.05), Cr–Ni (*r* = 0.76; *p* < 0.05), and Cr–Fe (*r* = 0.68; *p* < 0.05). Consequently, Fe, Ni, Cd, and Cr may be regarded as main marker elements of emissions from specialized steel production and metalworking in the Targoviste area.

Table 5 shows the grouping of children with respiratory issues in three spatial groups and the corresponding PM_2.5_ averages and associated heavy metal content in Targoviste City. The differences among the concentrations of heavy metals of the groups were small, which suggests that the impact of metallurgical activities affects larger areas. However, significant differences among the groups were observed for the PM_2.5_ averages that were recorded at the sampling points.

### Factorial analysis applied to the medical and air pollutant datasets

**Fig. 6:**
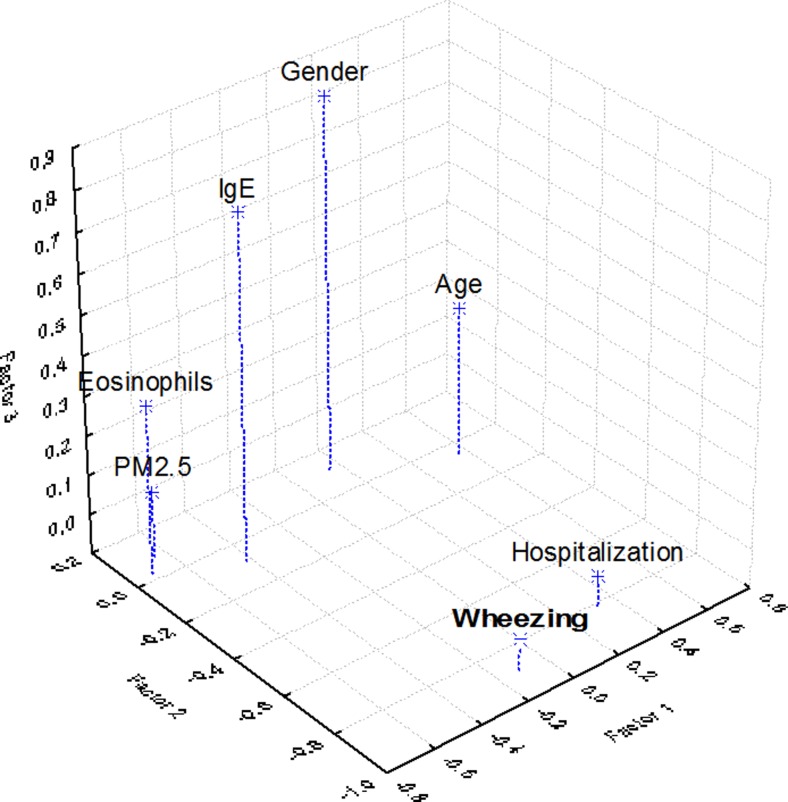
3D plot of loadings of the extracted factors using principal component analysis and Varimax normalized rotation

The relevant factor loadings (>0.55) were considered for each factor (Table 6). The rotated matrix showed that the eosinophil count, age of the child, and PM_2.5_ air pollution form the first factor (PC1), wheezing episodes and hospitalizations form the second factor (PC2), and IgE and gender form the third factor (PC3) (Fig. 6). These factors accounted for a cumulative variance of 64.5 % of the total variability in the dataset. The interpretation of the first factor loadings suggests that PM_2.5_ concentrations affect mainly small children having a major influence on eosinophils increasing, which are actively involved in inflammatory processes and allergy-like patterns of response. The health effect components, i.e., wheezing episodes and hospital admissions, showed high factor loadings. Consequently, factor analysis allowed the comparison of health effect estimates based on single pollutant metrics suggesting that air pollution with fine particulates may be a potential trigger of the asthma attacks leading to increases in hospital admissions, and the fact that the male subjects are potentially more susceptible to PM_2.5_ air pollution.

**Table 6:** Factor loadings (Varimax normalized) using principal component extraction (bolded loadings are >0.55)

Factor	Eigenvalue	Cumulative variance (%)	Wheezing episodes	Hospitalizations	IgE	Eosinophils	Age	PM_2.5_ air pollution	Gender
Factor 1	1.94	27.71	−0.11	0.33	−0.42	−0.71	0.57	−0.65	0.15
Factor 2	1.41	47.84	−0.89	−0.81	−0.08	0.03	−0.05	0.08	0.11
Factor 3	1.17	64.57	−0.02	−0.02	0.77	0.33	0.28	0.07	0.84

To support the FA results, an association between PM_2.5_ high concentrations and several physiological changes and clinical symptoms in children was observed in our study: alteration of lung function, symptoms of the upper and lower respiratory tracts, bronchial asthma, and rhinitis. Toxicological and clinical studies regarding the effects of combustion-derived particles showed that peak exposures of short duration (ranging from less than an hour to a few hours) lead to immediate physiological changes (WHO 2013). Hence, short-term exposure to peak and maximum levels of PM_2.5_ (Table 4) during the outdoor program of children in Targoviste has impacted the triggering of asthma exacerbations, especially in infants and preschoolers, which has increased the number of wheezing episodes and maintained elevated levels of allergic indicators (eosinophils and IgE) despite the use of controller medication for asthma.

## Conclusions

The monitoring results indicated high levels of fine particulates in the western and northwestern parts of Targoviste City, which were correlated with a high incidence of wheezing-related diseases in children up to 10 years old. The geolocation method that established the positioning of 72 children with high levels of IgE and eosinophils based on their residential address facilitated the spatial linking of PM_2.5_ and respiratory diseases. The study indicated that IgE level, eosinophil count, and wheezing episodes may be considered key indicators of the adverse effect of air pollution on children’s health. Exposure to PM_2.5_ impacted the asthma mechanism, especially in infants and preschoolers, which increased the number of wheezing episodes and the levels of allergic indicators.

PM_2.5_ contained the following heavy metals: Fe, Pb, Ni, Cd, and Cr. These metals were emitted from neighboring ferrous metallurgy processes, residential heating, and heavy traffic. The spatial distribution of children with elevated IgE and eosinophil levels did not correlate with any heavy metal concentrations because the aggregated levels that characterized each of the three groups were almost identical. Despite this lack of correlation, a high PM_2.5_ content with heavy metals and long-term exposure may exacerbate an already existing respiratory disease.

Additional detailed studies are required because the prevalence of asthma in Romania has increased in recent years to 7 % of the total child population, which has limited physical activity and increased school absenteeism.
